# Supplementary material for: Awareness of Malocclusion Is Closely Associated with Allergic Rhinitis, Asthma, and Arrhythmia in Late Adolescents
Source: Healthcare (Basel). 2020 Jul 12;8(3):209. doi: 10.3390/healthcare8030209 (PMC7551248; doi:10.3390/healthcare8030209)
Supplement: Supplementary file 1 [file healthcare-08-00209-s001.pdf]

Awareness of malocclusion

Do you have any difficulties chewing food due to malalignment of your teeth?	Yes	No
	☐	☐

Medical History

Please choose the presence or absence of the following disease(s)/disorder(s). When you chose the presence of a history for diseases/disorders, please write the specific name(s) of the disease(s)/disorder(s).

		Presence	Absence	Name of disease(s)/disorder(s)
1	Neuromasucular disease	☐	☐	
2	Cerebrovascular disease	☐	☐	
3	Coronary artery disease	☐	☐	
4	The other vascular disease	☐	☐	
5	Heart disease, arrhythmia, or abnormal electrocardiogram	☐	☐	
6	Hypertention	☐	☐	
7	Respiratory disease	☐	☐	
8	Esophagus, gastrointestinal disease	☐	☐	
9	Liver, gall bladder, pancreatic disease	☐	☐	
10	Renal disease	☐	☐	
11	Hyperuricemia, gout	☐	☐	
12	Diabetes mellitus	☐	☐	
13	Dyslipidemia	☐	☐	
14	Thyroid disease	☐	☐	
15	Endocrine disease	☐	☐	
16	Collagen disease	☐	☐	
17	Blood disease	☐	☐	
18	Malignant neoplasm	☐	☐	
19	Allergic disease	☐	☐	
20	Eye disease	☐	☐	
21	Otorhinolaryngologic disease	☐	☐	
22	Skin disease	☐	☐	
23	Bone, joint, muscle diseases	☐	☐	
24	Urologic disease, gynecological disease	☐	☐	
25	The other	☐	☐	

[Abe M et al., *Int J Environ Res Public Health* 2020, 17(12):E4290.]
